# Mindfulness-based music therapy for mental health in senior college students: a randomized controlled trial

**DOI:** 10.3389/fpsyg.2025.1649395

**Published:** 2025-10-29

**Authors:** Long Wang, Xiaoyan Zhang, Lei Pan, Rui Guo

**Affiliations:** School of Music, Hunan University of Technology and Business, Changsha, China

**Keywords:** mindfulness-based music therapy (MBMT), mental health, depression, anxiety, college students

## Abstract

**Background:**

Senior college students face a heightened risk of mental health problems, indicating the need for effective interventions. Mindfulness-based music therapy (MBMT) has been shown to improve mental health outcomes across various populations, yet evidence of its effectiveness in senior college students remains scarce. This randomized controlled trial aimed to test the efficacy of MBMT in improving mental health among senior college students.

**Methods:**

From September 2024 to December 2024, 400 senior college students were recruited and randomly assigned to the intervention group (*n* = 200) or the control group (*n* = 200). The intervention group received six weekly 40-min MBMT sessions, along with assigned homework, over 6 weeks. The primary outcomes included depression and anxiety, while the secondary outcomes included obsessive-compulsive symptoms, sleep quality, and resilience, which were assessed pre- and post-intervention.

**Results:**

A total of 181 students (adherence rate: 90.50%) in the intervention group and 197 students (adherence rate: 98.50%) in the control group completed the intervention and pre- and post-assessments. At baseline, the MBMT group and the control group showed no significant differences in sample characteristics and mental health indicators. Following the intervention, all mental health indicators in the MBMT group improved significantly and remained stable in the control group. The MBMT group demonstrated significantly lower levels of depression, anxiety, and obsessive-compulsive symptoms, as well as higher levels of sleep quality and resilience, compared to the control group post-intervention.

**Conclusion:**

MBMT is effective in decreasing depression, anxiety, and obsessive-compulsive symptoms and improving sleep quality and resilience among senior college students. Our findings suggest that MBMT may be incorporated into the curriculum for senior college students to enhance their mental health and general well-being.

## 1 Introduction

College students around the world are facing increasing mental health challenges, fueled by socioeconomic pressures, political and social instability, and technological overload (Salimi et al., 2021). According to the World Health Organization (WHO) survey across 21 countries, the 12-month prevalence of any DSM-IV/CIDI disorder was 20.3% among college students (Auerbach et al., 2016). A meta-analysis involving 100,187 individuals across 64 studies showed a pooled prevalence of 33.6% for depression and 39.0% for anxiety among college students (Li et al., 2022), which was much higher than the reported 12.9% for depression (Lim et al., 2018) and 4% for anxiety (GBD Results Tool, 2025) in the general population. Over the last decade, the incidence of mental health disorders among college students has increased significantly, which is further amplified by the COVID-19 pandemic and other societal factors (Wang et al., 2023). For instance, a study showed that the risk of depression and suicide ideation among college students increased by 50 and 100% in the post-pandemic period (Macalli et al., 2025). Senior college students are particularly at heightened risk for mental health problems in the final year due to a multitude of factors, including coursework culmination, academic pressure, post-graduation anxieties, financial strains, and career uncertainties (Zhang et al., 2024; Zou et al., 2018). Additionally, the transition into adulthood and the accompanying social pressures can contribute to increased stress and mental health challenges (Zhang et al., 2024; Zou et al., 2018).

Experiencing mental health problems can negatively affect college students’ academic performance, social relationships, and overall well-being, which may even lead to early dropout from school and inability to complete their degree (Sheldon et al., 2021; Hjorth et al., 2016; Mahdavi et al., 2023). Therefore, it is crucial to develop effective strategies to help them navigate these stressors and improve their mental health. A wide variety of intervention programs have been designed to enhance the mental health of college students, yet with varying degrees of effectiveness. Worsley et al. (2022) systematically reviewed a wide range of interventions aimed at improving college students’ mental health and well-being. These interventions included mindfulness-based interventions, psychological interventions, psychoeducation interventions, recreation programs, relaxation interventions, setting-based interventions, and stress management/reduction interventions (Worsley et al., 2022). They identified mindfulness practices among the most common and effective interventions (Worsley et al., 2022). Mindfulness involves being fully engaged in the current moment and paying attention to internal experiences, free from distractions or judgments, which can be cultivated through formal practices such as meditation, yoga, and body scan (Zhang et al., 2021).

A substantial body of evidence has demonstrated the beneficial effects of mindfulness practices in improving college students’ mental health and well-being (Bamber and Schneider, 2022; Bamber and Morpeth, 2019). Through fostering positive emotional and cognitive changes, mindfulness practices can contribute to reduced anxiety, depression, and stress, as well as enhanced self-regulation, self-efficacy, sleep quality, and quality of life (Bamber and Schneider, 2022; Bamber and Morpeth, 2019). Mindfulness practices are effective complementary tools for managing obsessive-compulsive disorder (OCD) symptoms and building resilience. Rather than trying to suppress intrusive thoughts and compulsive urges, mindfulness teaches individuals to observe them with non-judgmental awareness (Pseftogianni et al., 2023). Mindfulness helps break the obsessive-compulsive cycle through decentering, reducing compulsions, enhancing emotional regulation, and increasing self-awareness (Pseftogianni et al., 2023). Mindfulness also improves emotional tolerance, enhances cognitive flexibility, and cultivates self-compassion, all of which contribute to greater resilience (Liu and Zhou, 2022).

In addition, increasing evidence suggests that integrating mindfulness with other therapies can lead to greater overall benefits for mental health and general well-being than either approach alone (Chan et al., 2022). Particularly, combining mindfulness with music can have a synergistic impact on mental health, leading to enhanced emotion regulation, reduced stress, and improved psychological well-being (Hwang, 2023). Music can act as a valuable tool for focusing on the present moment, diverting negative thoughts, and inducing a sense of calm (Hwang, 2023). Additionally, the structural aspects of music, such as pitch, timbre, and tempo, can influence and shape emotional experiences, leading to a deeper understanding and acceptance of emotions and feelings (Chan et al., 2023). The integration of music and mindfulness, known as mindfulness-based music therapy (MBMT), can produce a synergistic effect on stress management and overall psychological well-being through dual activation of the neural and cardiac mechanisms associated with stress relief (Ramirez et al., 2025). Such a combination can create a robust foundation for personal growth and therapeutic exploration by overcoming avoidance, promoting psychological decentering, and increasing self-awareness (Chan et al., 2023). MBMT has demonstrated effectiveness in improving various mental health outcomes across multiple populations. For instance, MBMT has been shown to improve emotional regulation in blind older women (Chan et al., 2023) and decrease anxiety and stress in adolescents and young adults with cancer (Knoerl et al., 2022). It has also been shown to reduce nurse stress and work-related strain while improving their psychological well-being during the COVID-19 pandemic (Yildirim and Ciris Yildiz, 2022).

While general music therapy and mindfulness have been studied separately in college students generally, research has not sufficiently examined the specific effectiveness of MBMT for late-stage senior college students. This group faces unique stressors related to graduation, such as navigating job markets or graduate school applications, potential financial instability, and the transition from structured university life to independence (Magier et al., 2023). To fill in the research gap, we conducted a randomized controlled trial (RCT) to test the effectiveness of MBMT in improving mental health among senior college students. Our primary outcome was emotional distress, including depression and anxiety. Our secondary outcomes included sleep quality, obsessive-compulsive symptoms, and resilience.

## 2 Methods

### 2.1 Study design and participants

This was a 6-week, parallel-group, unblinded, randomized controlled trial conducted at Hunan University of Technology and Business, Changsha City, Hunan Province, China, from September 3, 2024, to December 12, 2024. The study protocol was approved by the Institutional Ethics Committee of Hunan Technology and Business University (Approval No. 2025-HUTB-PSY-02006). All participants provided written informed consent before the study. The study is reported in accordance with the Consolidated Standards of Reporting Trials (CONSORT) checklist.

The study participants were senior college students enrolled in a Music Appreciation elective course. The inclusion criteria were as follows: (1) full-time senior college students, (2) age ≥18 years old, and (3) having registered for the elective course of Music Appreciation. The exclusion criteria were as follows: (1) students with severe mental illness and suicidal ideation or behaviors; (2) students who were absent due to sickness, vacation, studying abroad, or other reasons during the study period; and (3) students who had received psychotropic medications or other therapies (e.g., mindfulness interventions, music therapy, and cognitive-behavior therapy) during the past 3 months. The attrition criteria were as follows: (1) participants in the intervention group who completed fewer than 2 MBMT sessions, (2) participants who voluntarily dropped the course or withdrew from the study, and (3) students who did not complete the pre- and post-survey questionnaires.

### 2.2 Sample size

The sample size was estimated using G-Power 3.1.9.7, based on two primary outcome indicators: depression, as assessed by the Zung Self-Rating Depression Scale (SDS), and anxiety, as assessed by the Zung Self-Rating Anxiety Scale (SAS). For this study, *α* was set at 0.05, the power was set at 0.95, assuming a medium effect size of 0.35 for both SAS and SDS. Considering an attrition rate of 15%, the required sample size was 200 for each group.

### 2.3 Randomization and allocation

All participants were fully informed of the study’s purpose, procedure, benefits, and risks at the first introductory class of the Music Appreciation course. After providing electronic informed consent, participants were randomly assigned to either the intervention group or the control group. A centralized computer system was used to ensure allocation concealment by managing the random assignment of participants through a secure, independent, and automated process. The statistician (Lei Pan) used pre-specified Stata code to generate random numbers, ensuring a 1:1 allocation of participants (StataCorp and Banks, 2015). The list containing student IDs for participants and their assigned groups was returned to the researcher (Xiaoyan Zhang). A total of 400 students were registered in the Music Appreciation course and were recruited, who were randomized into two groups: an intervention group (*n* = 200) that received MBMT alongside the course, and a control group (*n* = 200) that received the standard course content without MBMT.

### 2.4 Intervention

The intervention was described according to the Reporting Guidelines for Music-based Interventions checklist (Robb et al., 2025). The intervention was adapted from Lesiuk’s protocol, and the contents were based on four core mindfulness attitudes: non-judging, beginner’s mind, suspending judgment, and acceptance (Lesiuk, 2016). Non-judging refers to observing thoughts, feelings, and sensations without attaching labels or judgments to them. The Beginner’s mind is a concept that encourages viewing the world with an open, curious, and fresh perspective, as if for the first time, without being burdened by past experiences or judgments. Suspending judgment involves the willingness to try new things and listen without immediately judging or rejecting them, allowing for a deeper exploration and understanding of the experience. Acceptance is a mental practice that involves embracing the present moment without trying to change or resist it, focusing on what you can control instead of what you cannot. These four attitudes cultivate a state of openness and acceptance, which allows for a deeper connection to the present experience (Lesiuk, 2016). In addition, music can act as a tool to support these attitudes, ultimately helping individuals remain grounded in the moment (Lesiuk, 2016).

The interventionists included the instructor of the Music Appreciation course, a certified music therapist with 10 years of experience in music therapy, and a teacher assistant, a certified clinical psychologist with 5 years of experience in mental health counseling. The interventionists received standard uniform training on MBMT by attending a one-week workshop before the study. The intervention was delivered through the Music Appreciation course, and we designed six weekly MBMT sessions embedded in six music classes, with each session lasting approximately 40 min. Each MBMT session combined a variety of mindfulness practices (such as meditation, yoga, and mindful breathing) and music-related activities (such as listening to classical music, singing, imitating rhythms, and playing instruments). During these sessions, the participants were also guided to imagine themselves in specific scenarios, such as in a park or lying on the lawn, while concurrently focusing on their breathing. To enhance effectiveness, homework was assigned after each intervention to practice the session for 15 to 20 min daily. Further details of the themes and content of the intervention are shown in Table 1.

**Table 1:** Themes and contents of MBMT.

Session	Themes	Contents	Details	Homework
1	Introduction	Introduction to mindfulness Introduction to music therapy Introduction to mindfulness practices: meditation, yoga, and mindful breathing Introduction to music-related activities: listening to classical music, singing, imitating rhythms, and playing instruments.	The interventionists (instructor and teacher assistant) selected music based on Lesiuk’s protocol, personal experiences, and discussion with students.	Review the theory and framework of MBMT Choose one mindfulness practice and one music-related activity to practice for 15 min daily
2	Non-judging	Music Listening exercise with five different types of music Reflections on the music and linking to personal experiences	Beethoven, Moonlight Sonata, First Movement Flight of the Bumblebee (Erhu and Piano Versions) by Nikolai Andreivitch Rimsky-Korsakov Canyon (Guitar Version, Original Music) Forest, Joy The Mass	Listen to music for 15 min daily Write about at least one pleasant or unpleasant event daily
3	Beginner’s mind	Playing an instrument Singing familiar songs Reflections on their liking, sensory responses, and perceptions of their experience. Formally introduces a “beginner’s mind.”	Novel instruments include the mbira, ocean drum, pentatonic bars, and rain sticks. Participants explore the feel and timbre of these new instruments through touch and sound and discuss their enjoyment, sensory reactions, and subjective experiences.	Play an instrument and sing the familiar songs for 15 min daily Write about at least one non-music event daily, utilizing beginner’s mind.
4	Suspending judgment	Imitating and creating simple rhythms Designing multiple instrumental playing activities to train sustained concentration. Challenging settings to combat the adverse effects of “life and study pressure.” Reflections on feelings, thoughts, judgment	Imitating and creating simple rhythms on the big frame drum Playing simple harmonic changes on the xylophone Playing sustained patterns (bordun, repeated patterns) on the piano keyboard Playing familiar melodies on the keyboard according to number prompts.	Each student was given a maraca (sounds like a small maraca) to take home for rhythm practice for 15 min daily Practice the attitude of suspending judgment with daily events
5	Acceptance	Music-assisted relaxation Deep breathing practice Scripted imagery exercise Let go of negative experiences and emotions	The interventionists improvised on the piano while reading the script “Sending Thoughts Away on Clouds.” The participant imagined putting their worries into clouds and letting them float away in the sky. Background Music: 1. Daniel Kobialka, “Love Will Never Go Away” (Album: Beyond Embracing Dreams) 2. Whispers of Rain, Album: Nurturing Rain (Composer: Dan Gibson)	Practice music-assisted relaxation scripts for 15 min daily Practice the mindfulness attitude of “letting go” during the relaxation.
6	Conclusion	Revision of previous exercises Reflections on one’s thoughts, feelings, and mental state Conclusions		Practice previous practices and exercises Write about daily events and reflections

### 2.5 Control

The control group received the standard course content without MBMT but could access other forms of therapy at their own discretion, the same way as the intervention group.

### 2.6 Measures

Students completed paper-based questionnaires to assess their mental health at the beginning and end of the Music Appreciation course. The questionnaires included demographic information such as age, gender, major, and residence, and standard scales to assess depression, anxiety, obsessive-compulsive symptoms, sleep quality, and resilience.

#### 2.6.1 Primary outcomes

The Zung Self-Rating Depression Scale (SDS) was used to assess the frequency of experiencing depression symptoms over the past week (Zung, 1965). The SDS consists of 20 items, each rated on a 4-point Likert scale ranging from 1 “a little of the time” to 4 “most of the time.” The total raw score ranges from 20 to 80, which is then converted to index scores (25 to 100) by multiplying by 1.25. A higher index score indicates more severe depression symptoms, with a cut-off score of 50 or higher indicating the presence of depression. The SDS demonstrated good internal consistency in the current study, with a Cronbach’s alpha coefficient of 0.85.

The Zung Self-Rating Anxiety Scale (SAS) was used to assess the frequency of experiencing anxiety symptoms over the past week (Dunstan and Scott, 2020). The SDS consists of 20 items that cover a range of anxiety manifestations, including cognitive, autonomic, motor, and central nervous system symptoms. Individuals rate each item based on how much each statement applies to them, using a 4-point Likert scale ranging from 1 “a little of the time” to 4 “most of the time.” The total raw score ranges from 20 to 80, which is then converted to index scores (25 to 100) by multiplying by 1.25. A higher index score indicates more severe anxiety symptoms, with a cut-off score of 50 or higher indicating the presence of anxiety. The SAS demonstrated good internal consistency in the current study, with a Cronbach’s alpha coefficient of 0.83.

#### 2.6.2 Secondary outcomes

Obsessive-compulsive symptoms were assessed using the Obsessive-compulsive Symptom Scale (OSS) developed by Chinese scholar Qi et al. (2005). The OSS comprises 18 items under four dimensions: general obsessive thoughts (6 items), perfectionism (4 items), sexual obsession (3 items), and compulsive behavioral habits (5 items). Each item is rated on a 5-point Likert scale ranging from 1 “not at all” to 5 “very much.” The total score ranges from 18 to 90, with a higher score indicating more severe obsessive-compulsive symptoms. The OSS demonstrated good internal consistency in the current study, with a Cronbach’s alpha coefficient of 0.85.

Sleep quality was assessed using the Pittsburgh Sleep Quality Index (PSQI) to evaluate perceived sleep quality, sleep habits, and sleep disturbances over the past month (Buysse et al., 1989). The PSQI consists of 19 items, which are converted to 7 component scores: subjective sleep quality, sleep latency, sleep duration, habitual sleep efficiency, sleep disturbances, use of sleeping medication, and daytime dysfunction. Each component score ranges from 0 (no difficulty) to 3 (severe difficulty), and the total score ranges from 0 to 21, with a higher score indicating poorer sleep quality. A PSQI score of 5 or greater is considered indicative of poor sleep quality. The PSQI demonstrated good internal consistency in the current study, with a Cronbach’s alpha coefficient of 0.82.

Resilience was assessed using the Connor-Davidson Resilience Scale (CD-RISC) to evaluate an individual’s ability to cope with adversity and maintain positive functioning in the face of challenges (Chen et al., 2022). The CD-RISC consists of 25 items under five dimensions: tenacity and competence (8 items), trusting instincts (7 items), acceptance of change (5 items), control (3 items), and spiritual influences (2 items). Each item is rated on a 5-point Likert scale, ranging from 0 “not true at all” to 4 “true nearly all the time.” The total score ranges from 0 to 100, with a higher score indicating a higher level of resilience. The CD-RISC demonstrated excellent internal consistency in the current study, with a Cronbach’s alpha coefficient of 0.93.

#### 2.6.3 Acceptability and satisfaction

After the intervention, we further evaluated the intervention group’s perspectives of acceptability and satisfaction with MBMT using an Acceptability Scale (AS), which consists of seven items adapted from the Acceptability E-Scale (Carlson et al., 2001). Participants were asked to rate whether the intervention was easy, enjoyable, understandable, helpful, acceptable, and satisfying. Questions were rephrased to pertain to the evaluation of MBMT among college students. Each item is rated on a 5-point Likert scale ranging from 1”not at all” to 5 “very much.” The total score ranges from 7 to 35, with higher scores indicating greater acceptability and satisfaction with the intervention. The AS demonstrated excellent internal consistency in the current study, with a Cronbach’s alpha coefficient of 0.95.

### 2.7 Data analysis

All data were analyzed via STATA statistics (version 17.0). Continuous variables were expressed as means and standard deviations (SDs), while categorical variables were presented as numbers and proportions. The analysis used an intention-to-treat (ITT) approach. Since no more than five individuals for each measure at baseline had a single entry missing, indicating missing at random, multiple imputation (MI) was adopted for ITT analysis. Independent sample t-tests and χ2 tests were used to compare the baseline characteristics between the intervention and control groups, as well as between the study sample and the dropout sample. The intervention effects were assessed using linear mixed-effects models (LMMs) with time (pre/post) as a within-subject factor and group (MBMT vs. control) as a between-subject factor. The LMM accounts for the non-independence of repeated measures taken from the same individual by including both fixed and random effects. Main effects of group, time, and group × time interaction effects were examined. Cohen’s d was calculated as the effect size between groups and between pre-post test measurements, based on the ITT rule. Effect sizes were categorized as small (*d* = 0.2), medium (*d* = 0.5), and large (*d* = 0.8). All statistical tests were two-sided, with a *p*-value < 0.05 indicating statistical significance. For multiple group comparisons, the Holm-Bonferroni correction was performed to control for type I error.

## 3 Results

### 3.1 Participant recruitment and baseline characteristics

**Figure 1:**
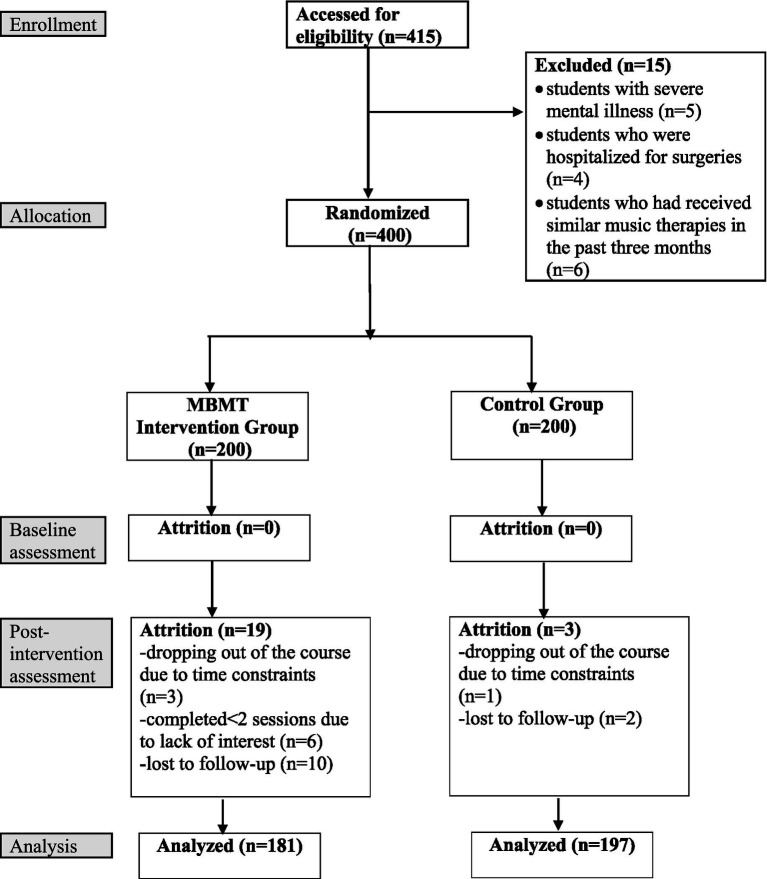
Consolidated Standards of Reporting Trials (CONSORT) flow diagram of participant recruitment and retention in the intervention.

A total of 415 students were registered for the Music Appreciation course, among whom 15 were excluded due to sickness-related absences, resulting in 400 students who were eligible and completed informed consent. They were randomly assigned to the intervention group (*n* = 200) and the control group (*n* = 200). Among the 400 participants, all completed the baseline assessment; however, 22 were excluded from the final analysis due to dropping out of the course (*n* = 4), completing fewer than two MBMT sessions (*n* = 6), or failing to complete the post-intervention assessment (*n* = 12). Finally, 378 participants were included in the final analysis, comprising 181 in the intervention group and 197 in the control group (Figure 1). The intervention group had a significantly higher attrition rate than the control group (9.50% vs. 1.50%, *p* < 0.001).

Table 2 shows the sample characteristics at baseline. The participants had a mean age of 20.15 ± 0.73 years, and 59.65% (238/400) were Female. Most students were from urban areas (65.50%), and a large proportion of them were majoring in music (39.50%), pursuing the same music degree. Their mean scores for depression, anxiety, obsessive-compulsive symptoms, sleep quality, and resilience were 38.87 ± 7.80, 36.34 ± 5.54, 35.56 ± 8.70, 3.60 ± 0.97, and 84.69 ± 13.52, respectively. There were no significant differences in baseline characteristics between the intervention group and the control group, and between the completed sample and the missing sample.

**Table 2:** Baseline characteristics of participants between the control group and the intervention group (*n* = 400).

Variables	Total sample (*N* = 400)	Control group (*n* = 200)	Intervention group (*n* = 200)	*p*
Age	20.15 ± 0.73	20.13 ± 0.74	20.16 ± 0.73	0.684
Gender				
Male	161 (40.35%)	77 (38.50%)	84 (42.21%)	0.450
Female	238 (59.65%)	123 (61.50%)	115 (57.79%)	
Residence				
Urban	262 (65.50%)	131 (65.50%)	131 (65.50%)	0.776
Rural	138 (34.50%)	69 (34.50%)	69 (34.50%)	
Major				
Music	158 (39.50%)	79 (39.50%)	79 (39.50%)	1.00
Design/Art	26 (6.50%)	16 (8.00%)	10 (5.00%)	
Foreign language	40 (10.00%)	20 (10.00%)	20 (10.00%)	
Public administration	24 (6.00%)	13 (6.50%)	11 (5.50%)	
Business administration	52 (13.00%)	27 (13.50%)	25 (12.50%)	
Computer	33 (8.25%)	17 (8.50%)	16 (8.00%)	
International business	37 (9.25%)	17 (8.50%)	20 (10.00%)	
Digital media engineering	30 (7.50%)	11 (5.50%)	19 (9.50%)	
Mental health indicators				
SDS	38.87 ± 7.80	38.80 ± 7.39	38.94 ± 8.21	0.858
SAS	36.34 ± 5.54	36.46 ± 5.24	36.22 ± 5.84	0.665
OSS	35.56 ± 8.70	35.99 ± 9.11	35.13 ± 8.28	0.321
General obsessive thoughts	12.63 ± 3.80	12.73 ± 3.83	12.52 ± 3.78	0.582
Perfection obsession	8.61 ± 3.03	8.85 ± 3.25	8.37 ± 2.78	0.113
Sexual obsession	4.82 ± 1.77	4.79 ± 1.91	4.86 ± 1.63	0.694
Compulsive behavioral habits	9.50 ± 3.06	9.62 ± 3.02	9.38 ± 3.10	0.424
PSQI	3.60 ± 0.97	3.58 ± 1.00	3.61 ± 0.94	0.772
CD-RISC	84.69 ± 13.52	84.62 ± 11.04	84.76 ± 15.64	0.915
Tenacity and competence	28.10 ± 5.26	28.04 ± 4.46	28.17 ± 5.97	0.813
Trusting instincts	22.80 ± 3.80	22.74 ± 3.16	22.87 ± 4.35	0.733
Acceptance of change	17.59 ± 3.17	17.63 ± 2.68	17.55 ± 3.60	0.813
Control	9.68 ± 2.19	9.71 ± 2.09	9.64 ± 2.29	0.750
Spiritual influences	6.52 ± 1.37	6.50 ± 1.28	6.54 ± 1.45	0.798

CD-RISC, Connor-Davidson Resilience Scale; OSS, Obsessive-compulsive Symptom Scale; PSQI, Pittsburgh Sleep Quality Index; SAS, Self-Rating Anxiety Scale; SDS, Self-Rating Depression Scale.

### 3.2 Primary outcomes

As shown in Table 3, the LMM revealed statistically significant group effects (*p* < 0.001 for all) and group-by-time interaction effects (*p* < 0.001 for all) for both depression (SDS) and anxiety (SAS). After the intervention, the MBMT group showed more substantial decreases in SDS (*β* = −2.48, *p* < 0.001) and SAS scores (*β* = −2.41, *p* < 0.001) than the control group, with intergroup effect sizes being 0.40 (95% CI 0.27–0.81) and 0.30 (95% CI 0.25–0.78), respectively.

**Table 3:** Between-group comparisons of changes in outcome variables over time.

Outcome and time	*β* (95% CI)	*p*	Cohen’s *d* (95% CI)	Group effect		Time effect		Group × time effect	
				Wald *χ*^2^ (*df* = 1)	*p*	Wald *χ*^2^ (*df* = 1)	*p*	Wald *χ*^2^ (*df* = 1)	*p*
SDS				40.36	<0.001	0.29	0.861	33.78	<0.001
Pre	−0.42 (−3.21, 2.25)	0.568	Reference						
Post	−2.48 (−0.99, −3.97)	<0.001	0.40 (0.27, 0.81)						
SAS				35.78	<0.001	0.49	0.501	11.43	<0.001
Pre	−0.55 (−2.88, 0.97)	0.256	Reference						
Post	−2.41 (−1.27, −3.55)	<0.001	0.30 (0.25, 0.78)						
OSS				62.49	<0.001	23.89	<0.001	58.89	<0.001
Pre	−0.87 (−0.85, 2.58)	0.321	Reference						
Post	−3.85 (−2.17, −5.53)	<0.001	0.76 (0.28, 0.92)						
GOT				54.44	<0.001	21.76	<0.001	42.45	<0.001
Pre	−0.21 (−0.54, 0.96)	0.582	Reference						
Post	−0.79 (−0.07, −1.51)	0.031	0.47 (0.14, 0.94)						
PO				42.56	<0.001	1.42	0.231	25.66	<0.001
Pre	−0.48 (−0.11, 1.07)	0.114	Reference						
Post	−1.07 (−0.49, −1.64)	<0.001	0.32 (0.17, 0.97)						
SO				39.95	<0.001	1.55	0.228	28.37	<0.001
Pre	0.07 (−0.42, 0.28)	0.694	Reference						
Post	−0.83 (−0.48, −1.18)	<0.001	0.68 (0.28, 0.95)						
CBH				49.94	<0.001	22.86	<0.001	36.99	<0.001
Pre	−0.25 (−0.36, 0.85)	0.425	Reference						
Post	−1.15 (−0.57, −1.74)	<0.001	0.75 (0.18, 0.91)						
PSQI				35.67	<0.001	0.58	0.453	27.98	<0.001
Pre	−0.02 (−0.21, 0.17)	0.838	Reference						
Post	−0.65 (0.45, 0.86)	<0.001	0.63 (0.16, 0.83)						
CD-RISC				74.67	<0.001	34.57	<0.001	60.66	<0.001
Pre	0.15 (−2.81, 2.52)	0.915	Reference						
Post	6.94 (4.44, 9.45)	<0.001	0.78 (0.47, 0.81)						
TC				63.49	<0.001	25.44	<0.001	48.86	<0.001
Pre	0.13 (−1.16, 0.91)	0.813	Reference						
Post	1.89 (0.93, 2.85)	<0.001	0.57 (0.17, 0.97)						
TI				65.95	<0.001	26.28	<0.001	56.88	<0.001
Pre	0.13 (−0.88, 0.62)	0.733	Reference						
Post	1.90 (1.20, 2.60)	<0.001	0.69 (0.28, 0.92)						
AOC				62.94	<0.001	0.45	0.522	48.97	<0.001
Pre	0.08 (−0.55, 0.70)	0.813	Reference						
Post	1.56 (0.94, 2.18)	<0.001	0.55 (0.44, 0.90)						
C				54.67	<0.001	16.39	<0.001	40.34	<0.001
Pre	0.07 (−0.36, 0.50)	0.750	Reference						
Post	0.75 (0.34, 1.16)	<0.001	0.65 (0.40, 0.85)						
SI				66.39	<0.001	0.39	0.540	57.35	<0.001
Pre	0.04 (−0.30, 0.23)	0.798	Reference						
Post	0.84 (0.57, 1.11)	<0.001	0.68 (0.34, 0.89)						

AOC, Acceptance of change; C, Control; CBH, Compulsive behavioral habits; CD-RISC, Connor-Davidson Resilience Scale; GOT, General obsessive thoughts; OSS, Obsessive-compulsive Symptom Scale; PO, Perfection obsession; PSQI, Pittsburgh Sleep Quality Index; SAS, Self-Rating Anxiety Scale; SDS, Self-Rating Depression Scale; SI, Spiritual influences; SO, Sexual obsession; TC, Tenacity and competence; TI, Trusting instincts.

### 3.3 Secondary outcomes

Table 3 also shows the LMM results for obsessive-compulsive symptoms (OSS), sleep quality (PSQI), and resilience (CD-RISC). Statistically significant group effects (*p* < 0.001 for all), time effects (*p* < 0.001 for all), and group-by-time interaction effects (*p* < 0.001 for all) were observed for both obsessive-compulsive symptoms and resilience. After the intervention, both groups showed significant changes in OSS (*p* < 0.001) and CD-RISC scores (*p* < 0.001). However, the MBMT group showed more substantial changes in OSS (*β* = −3.85, *p* < 0.001) and CD-RISC scores (*β* = 6.94, *p* < 0.001) than the control group, with intergroup effect sizes being 0.76 (0.28, 0.92) and 0.78 (0.47, 0.81), respectively. In addition, statistically significant group effect (*p* < 0.001) and group-by-time interaction effect (*p* < 0.001) were observed for sleep quality. The MBMT group showed a more substantial decrease in PSQI score (*β* = 0.65, *p* < 0.001) than the control group, with intergroup effect size being 0.63 (0.16, 0.83). Similar significant intervention effects were also observed in the subscales of OSS and CD-RISC, and the details are shown in Table 3.

### 3.4 Acceptability and satisfaction

Table 4 shows the intervention group’s feedback on the MBMT sessions as assessed by the AS. The participants expressed high levels of acceptability and satisfaction with the intervention, with the mean score of each item ranging from 4.84 ± 0.49 to 4.91 ± 0.43 (all ≥4).

**Table 4:** Intervention feedback using the item scores of the Acceptability Scale (AS).

Item	Mean
1. How easy was it for you to attend the MBMT sessions during the study?	4.87 ± 0.42
2. How understandable was the content presented in the MBMT sessions?	4.88 ± 0.41
3. How much did you enjoy participating in the MBMT sessions?	4.90 ± 0.42
4. How helpful were the MBMT sessions in managing emotional distress in your daily life?	4.90 ± 0.46
5. Was the frequency of the MBMT sessions acceptable?	4.91 ± 0.43
6. Was the length of each MBMT session acceptable?	4.85 ± 0.49
7. Overall, how would you rate your satisfaction with the MBMT sessions?	4.91 ± 0.43
Total scores	34.20 ± 1.36

MBMT, Mindfulness-based music therapy.

## 4 Discussion

### 4.1 Summary of the findings

Our study is the first of its kind to combine mindfulness and music therapy, innovatively integrating them into a selective course for senior students to enhance their mental health and overall well-being. Our results showed that although the intervention group and control group had comparable characteristics and mental health status at baseline, the intervention group demonstrated significant improvement in depression, anxiety, obsessive-compulsive symptoms, sleep quality, and resilience as compared to baseline and the control group. Our findings confirm the feasibility and effectiveness of MBMT in improving mental health among senior college students, which offers important implications for future mental health promotion programs on campus.

### 4.2 Feasibility and acceptability

Our study revealed a high adherence rate to interventions, with 90.50% of participants completing at least two sessions and both pre- and post-assessments. Additionally, all students who attended the sessions completed their homework, achieving a 100% completion rate. Homework is essential for helping students apply new mindfulness and music strategies to manage stress and anxiety in real-world settings. Integrating homework, such as mindful music listening and mindful instrument practice, into the MBMT intervention can reinforce skills learned in sessions, foster independent practice, and extend the therapeutic effects into daily life. The high adherence rate confirms that embedding MBMT sessions within selective college courses is a viable approach for promoting intervention compliance, with potential long-term sustainability. Integrating the intervention within the college curriculum offers a feasible and practical approach as it reduces barriers to access and promotes participation for college students who often juggle multiple academic and extracurricular demands. Further post-intervention assessments showed that participants expressed high levels of acceptance and satisfaction with the MBMT sessions. Such positive feedback provides further evidence of the practical application and effectiveness of the MBMT intervention. This finding suggests that MBMT may be scalable to a larger student population if effectively integrated into more courses or programs, thereby benefiting a greater number of students and improving their mental health and overall well-being.

However, it should also be noted that the intervention group had a significantly higher attrition rate than the control group (9.5% vs. 1.5%), also known as differential attrition (Bell et al., 2013). Multiple factors may affect participants’ adherence to the intervention, including academic overload, daily practice demands, unmet expectations, lack of perceived benefits, participant-investigator relationships, and other logistical challenges (Collins et al., 2022). For instance, the intervention may have imposed a higher academic burden than the control group’s standard activities by requiring additional time, effort, or lifestyle changes that were unsustainable for some students, leading to higher dropout rates. This finding suggests the need for proactive protocols to address known causes of attrition and minimize the risk of differential attrition. These strategies include maintaining clear communication, enhancing participant engagement, minimizing participant burden, providing transparent information, offering appropriate incentives, and collecting follow-up data for all participants (Elfeky et al., 2020).

### 4.3 Primary outcomes

Our study showed that MBMT significantly improved depression and anxiety symptoms of senior college students. This finding aligns with previous research demonstrating the effectiveness of mindfulness-based interventions in alleviating psychological distress across various clinical settings, indicating their potential for broader application (Chan et al., 2023; Knoerl et al., 2022; Yildirim and Ciris Yildiz, 2022). Previous research suggests that mindfulness-based interventions are more effective in reducing symptoms of depression and anxiety than non-evidence-based treatments, such as relaxation training, health education, supportive psychotherapy, and imagery or suppression techniques (Khoury et al., 2013; Hofmann and Gomez, 2017). These beneficial effects remained stable even after the follow-up period (Khoury et al., 2013; Hofmann and Gomez, 2017).

Furthermore, mindfulness-based interventions have demonstrated comparable efficacy with cognitive behavioral therapy (CBT), a well-established gold standard treatment for anxiety and depression (Khoury et al., 2013; Hofmann and Gomez, 2017). Previous meta-analyses showed that mindfulness-based interventions and CBT were equally effective in treating depression (Sverre et al., 2023) and anxiety (Li et al., 2021) at both post-intervention and follow-up. While MBMT and CBT share many similar characteristics and are both effective in managing depression and anxiety symptoms, they offer different approaches and aims. MBMT emphasizes acceptance and observation of thoughts to prevent relapses and reduce their negative impact, while CBT focuses on changing or restructuring negative thoughts and behaviors (Sverre et al., 2023; Li et al., 2021). Our findings will extend the available treatment options for depression and anxiety beyond the currently recommended psychotherapy, such as CBT, suggesting MBMT as a cost-effective alternative to address mental health problems (Sverre et al., 2023; Li et al., 2021). Our study has significant clinical implications, offering additional treatment approaches and empowering participants to choose the approach that best fits their needs, which aligns with the principle of personalized approaches to mental healthcare (Deif and Salama, 2021).

### 4.4 Secondary outcomes

Apart from emotional distress, MBMT has also been shown to decrease obsessive-compulsive symptoms and improve sleep quality and resilience. Obsessive-compulsive symptoms are characterized by persistent, unwanted thoughts (obsessions) that cause significant anxiety, often accompanied by repetitive behaviors (compulsions) aimed at reducing the anxiety (Woody et al., 2019). A meta-analysis showed that mindfulness-based interventions were effective in reducing obsessive-compulsive symptoms, with a small to medium pooled effect (SMD = −0.35) (Chien et al., 2022). MBMT can be an effective tool in managing obsessive-compulsive symptoms by fostering non-judgmental awareness of intrusive thoughts and reducing their perceived significance (Chien et al., 2022).

The beneficial effects of MBMT on sleep quality were congruent with a meta-analysis of 61 RCTs, which showed that mindfulness-based interventions were effective in improving sleep quality, with a large pooled effect (SMD = −0.794) (Yang et al., 2022). MBMT can reduce stress and anxiety that disrupt sleep, promote relaxation and easy transition to sleep, enhance body awareness, and release physical tension that interferes with sleep, all contributing to better sleep quality (Yang et al., 2022). Finally, the finding that MBMT significantly improved participants’ resilience also aligns with a meta-analysis of 57 RCTs, which showed a medium pooled effect size of mindfulness-based interventions in improving resilience (O’Connor et al., 2023). MBMT can help individuals develop a deeper understanding of their own mental and emotional processes, which is a core component of resilience (Christopher et al., 2018). In addition, MBMT encourages individuals to focus on the present and be flexible in their thinking and behavior, which is crucial for adapting to changing circumstances and building resilience (Christopher et al., 2018).

Notably, our study also showed that the control group experienced significant improvement in OSS and resilience, despite not participating in the MBMT intervention, demonstrating a control group effect. This finding highlights the potent indirect benefits of the Music Appreciation class through musical stimulation and social interaction. While the control group did not engage in active MBMT, passive engagement, such as listening to and learning about music, can still have a positive effect on brain and cognitive function, leading to improved resilience and decreased OSS (Thompson et al., 2023). Additionally, the social aspect of the Music Appreciation class can foster a sense of community, enhance interpersonal skills, and promote empathetic development, all of which contribute to improved mental health (Thompson et al., 2023). The improvement in the control group may complicate the interpretation of results and challenge the assumed effectiveness of the intervention. To address this issue, future research should incorporate more rigorous control conditions, such as enhanced blinding, repeated measurements, and waitlist control, to ensure that research findings are valid and reliable (Aday et al., 2022; Jensen et al., 2017).

### 4.5 Limitations

This study has several limitations. First, the sample was drawn from a single university and from a specific course (Music Appreciation), which may limit the external validity of our findings. The conclusions may not be generalizable to students at other institutions with different academic cultures, pedagogical approaches, and student demographics. To enhance the generalizability and robustness of these findings, future research should replicate this study across a diverse range of institutions, including public, private, and community colleges, as well as with students from various disciplines and socioeconomic backgrounds. Second, the intervention was delivered through a selective course of Music Appreciation, which may potentially serve as an informal music therapy-related intervention. Therefore, the control group should be framed as an active comparator rather than a true placebo or inactive control, and the effect sizes represent a comparison between the two, not a comparison of a treatment versus no treatment at all. Although the MBMT sessions differ from the music course content, combining MBMT and music courses can generate a synergistic effect that enhances both. Future studies may consider a multi-arm trial that includes an accurate placebo/no-intervention control to compare the effectiveness of music courses with or without MBMT, as compared to the control group, to gain a better understanding of the intervention effects. Third, we only collected data at pre- and post-intervention, and the absence of follow-up assessments is a key limitation. This can lead to unknown long-term efficacy, inability to identify delayed effects, risk of bias, limited validity, and a lack of insight into maintenance. Future RCTs with multiple assessments and longer follow-ups are needed to assess the long-term effectiveness of MBMT.

Fourth, this RCT was not blinded due to the nature of behavioral interventions. The lack of assessor blinding and the reliance on self-reported outcomes can introduce significant risks of bias, including observer bias, participant bias, social desirability bias, and recall bias, potentially leading to an overestimation of treatment effects. Future studies can implement strategies to mitigate bias, including blinding outcome assessors, using objective behavioral measures, and conducting sensitivity analyses. Finally, our study was conducted within a specific Chinese cultural context, and our results may not apply to other cultural groups, which could affect their validity and generalizability. Future studies should incorporate a cross-cultural perspective by comparing findings across different cultural groups to identify universal patterns versus culturally specific phenomena.

### 4.6 Practical and theoretical implications

Our study carries significant practical implications for implementing MBMT in academic environments, which should include comprehensive facilitator training and adapting the format to fit the institutional context, such as hybrid delivery. Facilitators should have an established personal mindfulness practice (lasting one to 2 years) with a deep, personal understanding of mindfulness to guide others more effectively. Facilitators should complete certification programs that blend online learning, in-person intensives, and individualized guidance with a focus on teaching skills, curriculum design, inclusivity, and trauma-informed care. Ongoing professional development is also necessary to ensure that facilitators remain current with research and maintain their professional practice. In addition, a hybrid format, which combines in-person and online instruction, can make MBMT more accessible and flexible for a diverse academic community by increasing accessibility, accommodating diverse schedules, and enhancing learning opportunities.

Apart from practical implications, our study also highlights the theoretical contribution of MBMT as a conceptual framework that moves beyond simply demonstrating its effectiveness as an intervention. It presents a multimodal and synergistic conceptual framework. It proposes a new, integrated model for combining music and mindfulness to produce a synergic effect that is more powerful than the sum of each component. This framework has important implications for both research and practice. Future research based on the framework should focus on the synergistic interactions between modalities, which can better inform and optimize MBMT interventions.

## 5 Conclusion

Our study shows that the 6-week MBMT intervention embedded in college curriculum is both feasible and effective in reducing depression, anxiety, and obsessive-compulsive symptoms, while improving sleep quality and resilience among senior college students. Our findings offer an innovative, cost-effective approach by combining mindfulness practice and music therapy into routine classes to enhance the mental health and overall well-being of college students. While the study provides valuable insight into the short-term effects of MBMT intervention within a single cultural context, the absence of a long-term follow-up across diverse populations may limit the generalizability and sustainability of these effects. Future multicenter or international studies with long-term follow-up phases are necessary to further verify our findings.

## Data Availability

The raw data supporting the conclusions of this article will be made available by the authors, without undue reservation.
